# Les facteurs associés à l'infection au cours de la polyarthrite rhumatoïde

**DOI:** 10.11604/pamj.2013.16.35.2571

**Published:** 2013-10-01

**Authors:** Nessrine Akasbi, Latifa Tahiri, Ghita Sqalli Houssaini, Taoufik Harzy

**Affiliations:** 1 Service de Rhumatologie CHU Hassan II, Fès, Maroc

**Keywords:** Polyarthrite rhumatoïde, infections, facteurs associés, rheumatoid arthritis, infections, associated factors

## Abstract

Les complications infectieuses sont redoutables au cours de la polyarthrite rhumatoïde (PR). Le but de notre étude est d'estimer leur fréquence et de déterminer les facteurs associés à l'infection chez ces patients. Il s'agit d'une étude rétrospective incluant les cas de PR établis recensés entre 2007 et 2011 au service de rhumatologie au CHU Hassan II de Fès au Maroc. Nous avons inclu 164 patients atteint de PR, l’âge moyen des patients était de 47,9 ans, avec une prédominance féminine (137 F/27H). La fréquence des infections dans notre série était de 26,2%, dominées par les infections urogénitales (22 cas), pleuro pulmonaires (11 cas) dont 2 cas de tuberculose pulmonaire et un cas d'infection H1N1, 3 cas d'infections cutanées et 4 cas d'arthrite septiques. Dans notre série 127 patients étaient sous corticothérapie orale, 147 patients étaient sous méthotrexate, 25 patients étaient sous rituximab et 8 patients étaient sous tocilizumab. Dans notre étude, les facteurs associés à la survenue d'infection étaient l’âge avancé (p= 0,02), une CRP élevée (p= 0,04) et une dose de corticothérapie - 7.5 mg/j (p= 0,03). Notre étude a mis en évidence certains facteurs associés à la survenue d'une infection au cours de la PR. En connaissant ces facteurs, il faut instaurer une surveillance particulière pour améliorer la qualité de prise en charge.

## Introduction

La polyarthrite rhumatoïde (PR) est le rhumatisme inflammatoire chronique le plus fréquent. Elle peut conduit à des destructions ostéoarticulaires sévères. La PR est responsable à long terme d'une augmentation de la morbidité et de la mortalité due essentiellement à une augmentation du risque infectieux. Ainsi, les patients atteints de PR sont particulièrement sensibles aux infections opportunistes ou sévères. Quelques études ont déjà montré que le risque de mortalité lié aux infections était augmenté dans la PR par rapport aux patients non atteints de PR [1–3], cependant d'autres études n'ont pas retrouvé de différence [4, 5]. L'augmentation du risque infectieux chez les patients atteints de PR pourrait être liée aux désordres immunologiques associés à la PR elle-même, ou aux mécanismes d'action des médicaments destinées à moduler ou à supprimer le système immunitaire. L'objectif de notre travail était d'estimer la prévalence des infections au cours de la PR et de déterminer les facteurs associés à l'infection au cours de la PR. La prise en charge de ces patients passe par la détection de ces facteurs afin d'améliorer la qualité de prise en charge des patients infectés.

## Méthodes

Il s'agit d'une étude rétrospective incluant tous les cas de polyarthrite rhumatoïde établis entre 2007 - 2011 au service de Rhumatologie au CHU Hassan II de Fès. Il s'agit soit une PR ancienne donc diagnostiquée selon les critères de l'ACR 1987 [6] ou récente et donc diagnostiquée selon les critères ACR EULAR 2010 [7].

Le recueil de données a été réalisé à partir d'une fiche d'exploitation contenant des données sociodémographique (l’âge, le sexe, les comorbidités), des données cliniques (la durée de l’évolution de la PR, l'activité de la PR évaluée par le disease activity index 28 Vitesse de sédimentation (DAS28 Vs), les manifestations extra-articulaires), des données paracliniques (le bilan inflammatoire: la vitesse de sédimentation (Vs), la C réactive protéine (CRP) et le bilan immunologique: facteur rhumatoïde (FR), anti-cyclic citrullinated peptide antibodies (anti CCP), des données thérapeutiques: la corticothérapie, le traitement de fond classique et les biothérapies. La confirmation de l'infection était basée sur une sérologie positive (hépatite B,C, HIV...)< 0,05 a été considéré comme significatif.

## Résultats

On a inclus 164 patients atteints de PR. L’âge moyen de nos patients était de 47.9 ans ± 11.9 (20.80). Ainsi, l’âge moyen de la population ayant développé une infection était de 50.8 ans, alors qu'il était de 47 ans chez la population non atteinte d'infection, cette association était statistiquement significative. On a noté une nette prédominance féminine avec un sexe ratio de 5 F/1H, (137 femmes pour 27 hommes). Dans notre population, 113 patients (68.9%) n'ont eu aucun antécédent pathologique notable, alors que, 20 patients étaient diabétiques, 6 patients étaient tabagiques chroniques.

La durée moyenne de l’évolution de la PR était de 8 ans ± 7 (1, 30 ans). Concernant les manifestations extra articulaires de la PR, on a noté 8 cas de nodules rhumatoïdes, 28 patients ont eu une atteinte cardiovasculaire à type d'hypertension artérielle ou de cardiopathie ischémique. On a eu un seul cas de vascularite rhumatoïde cutanée avec un ulcère de jambe, 46 patients ont développé une anémie inflammatoire qui s'est corrigée après un traitement de fond bien conduit, 23 patients ont eu une atteinte pulmonaire rhumatoïde à type de pneumonie infiltrante diffuse, de nodules rhumatoïdes pulmonaires ou de fibrose pulmonaire, 38 patients avaient un syndrome sec, dont 7 souffraient d'un goujerot sjogren retenu selon les critères de l'American European Consensus Group (AECG) [8], 7 patients ont eu des manifestations neurologiques à type de luxation atloido axoidienne chez 5 patients, un syndrome de canal carpien chez un patient et une polyneuropathie périphérique sensitivomotrice au niveau des membres inférieurs dans un cas.

Concernant le bilan inflammatoire réalisé au moment de l'infection, la moyenne de la vitesse de sédimentation (Vs) chez nos patients était de 51.6 mm/h ± 32.2, (2.136), la moyenne de la CRP chez nos patients était de 37.3 mg/l ± 52.3 (1.350). Concernant le résultat du facteur rhumatoïde réalisé au moment du diagnostic de la PR, 115 patients (70%) étaient séropositifs pour le facteur rhumatoïde, alors que 49 patients (30%) étaient séronégatifs. Seulement 127 de nos patients avaient bénéficié d'un dosage d'anticorps anti CCP, 110 de nos patients avaient des anti CCP positifs, alors que 17 patients avaient des anti CCP négatifs. L'activité moyenne de la PR (exprimée en DAS 28 Vs) chez nos patients était de 5.4 ± 1.5 (1-9.1).

Chez notre population, 127 patients (77.4%) avaient reçu une corticothérapie à long cours, avec une dose moyenne de 10.5 mg /jour ± 4.85 mg, (5.20) au moment du diagnostic de l'infection. Parmi ces patients, 91 (soit 71.65%) avaient une dose qui dépassait 7.5 mg/jour au moment de l'infection. La durée moyenne de prise de corticoïdes était de 4 ans, 41 patients (soit 32.3%) ont eu une durée de corticothérapie ‘ 5 ans.

Dans notre population, 156 patients ont reçu un traitement de fond, 147 patients ont reçu du méthotrexate (MTX), en monothérapie chez 143 patients, associé à la salazopyrin (SZP) chez 2 patients, associé à l'anti paludéen de synthèse (APS) chez 1 patient. Sept patients ont reçu la salazopyrin seul et 2 ont reçu le leflunomide seul (Tableau 1). La dose moyenne du méthotrexate chez nos patients était de 15.2 mg/semaine, avec ±3.4 (7.5, 25mg) au moment du diagnostic de l'infection. La durée moyenne de du MTX était 2 ans ± 1.4 (1- 7 ans).

**Tableau 1:** Différents traitements de fond classique utilisés chez nos patients

Médicament	Nombre de patients	%
**Méthotrexate (MTX)**	143	91.66
**Salazopyrin**	7	4.48
**Leflunomide**	2	1.28
**MTX + SZP**	2	1.28
**MTX + nivaquine**	1	0.64
**MTX + SZP + nivaquine**	1	0.64

Dans notre étude, 25 patients ont reçu du Rituximab à la dose de 1g en perfusion renouvelé après deux semaines, 21 patients en association avec le méthotrexate et 4 patients en monothérapie seul. Il y avait 8 patients qui ont été traités par du Tocilizumab à la dose de 8 mg/kg chaque 4 semaine. Dans notre travail aucun patient n'a été traité par un anti TNF’. Compte tenu que notre pays est une zone d'endémie tuberculeuse, la société marocaine de rhumatologie (SMR) recommande le Rituximab en première intention si PR avec intolérance ou échec du MTX à dose efficace tolérée pendant 3 mois de traitement ou si PR active et évolutive avec un DAS 28 > 5.2 ou > 3.2 avec une corticodépendance et des lésions d’évolutivité structurale.

Dans notre étude la prévalence des infections au cours de la PR était de 26.2% soit 43 cas pour tout type d'infection confondu. Nous avions colligé 22 cas d'infection urogénitale à type de cystite aux bacilles gram négatif notamment l'Escherichia coli qui ont bien évolué sous traitement antibiotique en ambulatoire, 11 cas d'infection pulmonaire dont 7 cas d'infection respiratoire aigue banale traités en ambulatoire, 2 cas de tuberculose pulmonaire, un cas de sepsis sur infection pulmonaire et un cas d'infection par H1N1, ces deux derniers ont nécessité une prise en charge aux soins intensifs, 4 cas d'arthrite septique qui ont bien évolué sous antibiotique et drainage arthroscopique, 3 cas d'infection virale B chronique mis sous traitement anti virale B, et 3 cas d'infection cutanée à type de cellulite faciale et d'abcès des parties molles. A noter qu'aucun cas d'infection sur cathéter dans le cadre de traitement par des biothérapies, qu'aucun cas d'infection grave nécessitant une hospitalisation n'a été retrouvé chez les patients sous biothérapie, et que ces patients n'ont pas présenté des épisodes infectieux récidivants.

En analyse multivariée (Tableau 2), les facteurs associés à la survenue d'infection au cours de la polyarthrite rhumatoïde dans notre étude étaient l’âge avancé, une dose de corticothérapie ≥ 7.5 mg/jour et l’élévation de la CRP, à noter que La relevance clinique de ce sur risque d′infection à 1.01 (IC 1.01 -1.02) lié à la CRP est discutable.

**Tableau 2:** Facteurs associés à l'infection chez nos patients atteints de la PR en analyse multivariée

Paramètre	Odd ratio	IC= 95%	p
Age	1.5	1.0-1.11	0.02
Sexe H/F	0.4	0.06-2.3	0.3
Comorbidités	1.7	0.4- 8	0.45
Diabète	0.5	0.07-3	0.45
Manifestations extra articulaires	0.4	0.1-1.5	0.18
Vs [mm/h]	0.99	0.97-1.01	0.8
CRP [mg/l]	1.01	1.0-1.02	0.04
Durée de PR	0.95	0.8-1	0.28
FR positif	1.07	0.3-3.5	0.9
Durée de corticothérapie [CO]	1.1	0.9-1.2	0.16
Dose de CO ≥ 7.5mg/j	9	1.2-66.5	0.03
Dose MTX	0.4	0.009-18	0.65

## Discussion

La polyarthrite rhumatoïde est le rhumatisme inflammatoire chronique le plus fréquent. Elle touche 0,5 à 1% des individus de la population générale. Elle prédomine chez la femme et se caractérise par une inflammation chronique et une destructrice de la membrane synoviale [9]. Le risque de mortalité lié aux infections est très important au cours de la PR, ces infections constituent la troisième cause de mortalité après les complications cardio-pulmonaires.

La fréquence accrue des infections au cours de la PR est expliquée d'une part par les désordres immunologiques et inflammatoires de la maladie elle-même notamment la présence de lymphocytes B anormales et de lymphocytes T déficientes et d'autre part aux mécanismes d'action des médicaments qui augmentent considérablement ce risque en diminuant les défenses de l'organisme [1]. En ce qui concerne la fréquence des infections au cours de la PR, la cohorte longitudinale rétrospective de Rochester [10] a comparé la fréquence des infections chez les patients atteints de PR diagnostiquée entre 1955 et 1994 par rapport à un groupe de patient non atteint de PR, elle avait montré une augmentation significative de l'incidence des infections dans le groupe PR (19,64/100 patients/an versus 12,87/100 patients/an), ainsi qu'une augmentation de l'incidence des infections sévères nécessitant une hospitalisation dans le même groupe (9,57/100 patients/an versus 5,09/100 patients/an).

Les infections peuvent intéresser tous les sites avec une fréquence élevée pour les infections ostéo-articulaires: arthrites septiques RR: 10,63 (95% IC: 6,12-73,71), les infections urogénitales et les infections cutanées et des tissus mous RR: 3,28 (95% IC: 2,67-4,07) [4].

Dans la littérature, certains facteurs peuvent prédire la survenue de l'infection au cours de la PR, il s'agit de l’âge avancé, le sexe masculin, certaines comorbidités incluant les pneumopathies chroniques, le diabète, le tabagisme, l’éthylisme et la neutropénie, des facteurs liés à l'activité de la PR comportant les manifestations extra-articulaire, les nodules rhumatoïdes, le facteur rhumatoïde et le syndrome inflammatoire, le recours à la chirurgie orthopédique, l'immobilisation, et un traitement par corticothérapie, concernant les traitements de fond classique, les résultats sont controversés [11].

Les facteurs retrouvés dans notre série sont l’âge avancé, l’élévation de la CRP et la dose de corticothérapie qui dépasse 7.5 mg/j, tout en rappelant que la relevance clinique de ce sur risque d′infection à 1.01 (IC 1.01 -1.02) lié à la CRP est discutable. Dans notre l’âge moyen des patients atteints d'infection était de 50.8 ans. L’âge avancé était un facteur associé à la survenue d'infection (p=0,02). Nos patients avaient 1.5 fois le risque de développer une complication infectieuse au cours de l’évolution de leur maladie.

Il est admis que la corticothérapie utilisée comme traitement symptomatique de la PR est un facteur de risque d'infection. La dose de corticoïdes associée au risque infectieux n'est pas connue, toutefois le risque existe même pour les faibles doses (5 mg/j [12]. Une étude comparant 112 patients atteints de PR traités par glucocorticoïdes à un groupe de PR non traités par glucocorticoïdes a montré que le risque infectieux est plus élevé dans le groupe recevant les corticoïdes avec un RR à 8 (95% IC (1-64), p: 0,05) [13]. Dans notre étude une dose de corticothérapie ≥7.5 mg/j était associée à un risque de survenue d'infection. Les patients ayant reçu une corticothérapie avaient 9 fois le risque de développer une infection. Les mécanismes qui expliquent l'immunodépression chez les patients recevant une corticothérapie sont surtout la survenue de perturbations de l'immunité cellulaire avec réduction significative des taux de lymphocytes T et des cytokines synthétisées, notamment l'interféron gamma, la diminution de la fonction monocytaire et la suppression non spécifique des cytokines pro-inflammatoires [14]. Par conséquent, une exposition de courte durée ou l'utilisation d'une alternative thérapeutique peuvent diminuer ce risque infectieux. D'où l'intérêt de prescrire une corticothérapie à dose minimale efficace pour une courte durée et introduire un traitement de fond le plus rapidement possible qui permettra de contrôler la maladie et induire une rémission le plus précocement possible avec moins de complications infectieuses.

Dans notre travail, ni la dose, ni la durée de traitement par MTX n’était associé à la survenue d'infection. Les études dans la littérature ont montré que les infections au cours du traitement par méthotrexate sont non seulement fréquentes mais sévères, mais cette association reste non claire [15–16]. Dans une étude prospective sur 12 mois incluant 77 patients souffrant de PR recevant du MTX et 151 patients porteur de PR ne prenant pas le MTX, a conclu que la fréquence des infections était supérieure chez les patients traités par MTX (62 vs 47%) [17].

Concernant les traitements biologiques nous connaissons le risque infectieux potentiellement sévère lié à l'utilisation des antiTNF’. Il s'agit des infections à germes pyogènes, opportunistes, et surtout mycobactériens notamment la tuberculose puisque le relarguage du TNF’ est primordial pour la réponse aux infections mycobactériennes [18]. Dans notre travail aucun patient n'a été traité par un anti TNF’. Les essais cliniques étudiant le rituximab dans la PR retrouve que les taux d'infections observés sous l'association Rituximab-méthotrexate sont très voisins de ceux observés sous placebo-méthotrexate avec cependant une augmentation statistiquement non significative du taux d'infections sévères [19–20]. Dans notre travail, seulement 4 patients sous Rituximab ont développé une infection bénigne qui a bien évolué sous simple antibiothérapie et tous étaient sous l'association Rituximab et MTX. Mais l'association n’était pas statistiquement significative. Il est judicieux d'appliquer les recommandations pré thérapeutiques pour diminuer le risque d'infection chez les patients atteints de PR sous Rituximab [21]. Le traitement par l'inhibition de l'IL-6, le tocilizumab, est susceptible d'interférer avec les capacités de défense anti-bactérienne et de favoriser la survenue et la réactivation de pathologies infectieuses. Le taux d'infections, principalement d'infections graves, est stable, et proche des chiffres observés avec les autres biothérapies [22]. Dans notre étude aucun cas d'infection parmi les 8 patients traités par tocilizumab n'a été colligié. A travers cette étude un certain nombre de suggestions sont à proposer: L'infection doit être systématiquement recherchée. Il faut agir sur les facteurs associés à la survenue d'infection au cours de la PR: sur la poussée inflammatoire de la PR par un traitement symptomatique efficace. Il faut une bonne gestion de la corticothérapie avec une dose minima efficace pendant une courte durée et un traitement de fond efficace le plus rapidement possible. Il faut instaurer une surveillance rigoureuse, stricte et rapprochée avec une implantation de stratégie préventive.

Vu le petit nombre de notre population étudiée, l’étude doit continuer de façon prospective dans plusieurs centres hospitaliers et sur une large population de patients atteints de polyarthrite rhumatoïde afin d'obtenir des résultats représentatifs et plus significatifs.

## Conclusion

Bien que peu fréquente dans notre série, l'infection au cours de la polyarthrite rhumatoïde devra être systématiquement recherchée car elle peut être grave et augmenter la morbi-mortalité non seulement du fait des perturbations immunologiques inhérentes à cette maladie mais aussi du fait des médicaments immunosuppresseurs notamment les corticoïdes et les thérapeutiques de fond.

Nos résultats concordent avec ceux de la littérature en ce qui concerne l'augmentation de la fréquence des complications infectieuses au cours de la PR et qui sont favorisées par la corticothérapie en premier, l’âge avancé, et l'inflammation chronique. A noter qu'aucun cas d'infection grave nécessitant une hospitalisation n'a été retrouvé chez nos patients sous biothérapie.
